# FGF1 C-terminal domain and phosphorylation regulate intracrine FGF1 signaling for its neurotrophic and anti-apoptotic activities

**DOI:** 10.1038/cddis.2016.2

**Published:** 2016-02-04

**Authors:** E Delmas, N Jah, C Pirou, S Bouleau, N Le Floch, J-L Vayssière, B Mignotte, F Renaud

**Affiliations:** 1 Laboratoire de Génétique et Biologie Cellulaire, EA4589 Université de Versailles St Quentin en Yvelines (UVSQ), Ecole Pratique des Hautes Etudes (EPHE), UFR des Sciences de la Santé Simone Veil, Montigny-Le-Bretonneux 78180, France

## Abstract

Fibroblast growth factor 1 (FGF1) is a prototypic member of the FGFs family overexpressed in various tumors. Contrarily to most FGFs, FGF1 lacks a secretion peptide signal and acts mainly in an intracellular and nuclear manner. Intracellular FGF1 induces cell proliferation, differentiation and survival. We previously showed that intracellular FGF1 induces neuronal differentiation and inhibits both p53- and serum-free-medium-induced apoptosis in PC12 cells. FGF1 nuclear localization is required for these intracellular activities, suggesting that FGF1 regulates p53-dependent apoptosis and neuronal differentiation by new nuclear pathways. To better characterize intracellular FGF1 pathways, we studied the effect of three mutations localized in the C-terminal domain of FGF1 (i.e., FGF1^K132E^, FGF1^S130A^ and FGF1^S130D^) on FGF1 neurotrophic and anti-apoptotic activities in PC12 cells. The change of the serine 130 to alanine precludes FGF1 phosphorylation, while its mutation to aspartic acid mimics phosphorylation. These FGF1 mutants kept both a nuclear and cytosolic localization in PC12 cells. Our study highlights for the first time the role of FGF1 phosphorylation and the implication of FGF1 C-terminal domain on its intracellular activities. Indeed, we show that the K132E mutation inhibits both the neurotrophic and anti-apoptotic activities of FGF1, suggesting a regulatory activity for FGF1 C terminus. Furthermore, we observed that both FGF1^S130A^ and FGF1^S130D^ mutant forms induced PC12 cells neuronal differentiation. Therefore, FGF1 phosphorylation does not regulate FGF1-induced differentiation of PC12 cells. Then, we showed that only FGF1^S130A^ protects PC12 cells against p53-dependent apoptosis, thus phosphorylation appears to inhibit FGF1 anti-apoptotic activity in PC12 cells. Altogether, our results show that phosphorylation does not regulate FGF1 neurotrophic activity but inhibits its anti-apoptotic activity after p53-dependent apoptosis induction, giving new insight into the poorly described FGF1 intracrine/nuclear pathway. The study of nuclear pathways could be crucial to identify key regulators involved in neuronal differentiation, tumor progression and resistances to radio- and chemo-therapy.

The fibroblast growth factor 1 (FGF1) is one of the 22 members of the FGF family.^1^ Most FGFs are secreted and mediate their activity through FGF receptors (FGFR1–4) located at the plasma membrane, which induce Ras (rat sarcoma)/mitogen-associated protein kinases, PI3K (phosphotidylinositide 3-kinase)/AKT and phospholipase C *γ* pathways.^2, 3^ However, the fate of all FGF members is not always to be secreted. In particular, FGF1, FGF2, one FGF3 isoform and FGF11–14, which do not contain any secretion peptide signal, are not secreted in physiological conditions and mediate their activity by intracrine pathways. Most of these intracrine factors contain one or more nuclear localization sequences (NLS), which regulate their nuclear translocation, a process required for their activities.^4, 5, 6, 7^ For example, FGF1 lacks a secretion peptide signal but contains a NLS (KKPK) and acts mainly in an intracellular and nuclear manner.^4, 8^ Intracellular FGF1 is a neurotrophic factor for various neuronal cells both *in vitro* and *in vivo.*^9, 10, 11, 12, 13^ It also activates DNA synthesis,^4, 14, 15^ exerts an anti-apoptotic activity,^11, 16, 17^ and its overexpression correlates with tumor development and chemotherapy resistance.^18, 19, 20^

We have previously shown that intracellular FGF1 inhibits p53-dependent apoptosis by an intracrine pathway in both fibroblasts and neuronal-like cells.^16, 17^ Using fibroblasts, we have shown that *fgf1* is a repressed target gene of p53 and that overexpression of FGF1 decreases both the pro-apoptotic and the anti-proliferative activities of p53. In these cells, intracellular FGF1 mediates its activities by two mechanisms of action: (i) FGF1 increases MDM2 (mouse double minute 2) expression, which leads to p53-degradation; (ii) FGF1 decreases p53-dependent transactivation of *bax*, which encodes a pro-apoptotic B-cell lymphoma 2 family (Bcl-2) member protein required for p53-dependent apoptosis in fibroblasts.^16^

FGF1 is highly expressed in central and peripheral nervous systems and its neurotrophic and anti-apoptotic activities are well documented *in vitro* and *in vivo.*^11, 13, 21, 22, 23, 24^ However, the mechanisms of action of FGF1 for these activities remain to be characterized. Thus, we have pursued our study in neuronal-like PC12 cells. We showed that FGF1 inhibits both p53-dependent and serum free-medium-induced apoptosis and exerts its neurotrophic activity by an intracrine pathway in PC12 cells.^17^ Using the FGF1^ΔNLS^ mutant that is deleted of its NLS, we showed that the FGF1 nuclear translocation is required for both its anti-apoptotic and neurotrophic activities. Intracellular FGF1 regulates p53-dependent apoptosis by decreasing p53 phosphorylation on serine 15, which is a marker of its activation, p53-dependent transactivation of *puma*, which encodes a pro-apoptotic Bcl-2 family member required for p53-dependent cell death in PC12 cells, and caspase-3 activation. All these effects of FGF1 cooperate to protect PC12 cells from p53-dependent apoptosis. The FGF1^ΔNLS^ mutant, which only presents a cytosolic localization, has no effect on p53-dependent apoptosis in PC12 cells. Altogether, these data suggest that FGF1 regulates p53-dependent apoptosis and neuronal differentiation by new intracrine/nuclear pathways.^17^

The aim of the present paper is to progress in the characterization of these nuclear pathways. For this purpose, the activity of new mutated forms of FGF1 in PC12 cells was examined. We studied the effect of three mutations located in the C terminus of FGF1 (i.e., FGF1^K132E^, FGF1^S130A^ and FGF1^S130D^) on its intracellular neurotrophic and anti-apoptotic activities.

Previous studies using recombinant FGF1^K132E^ showed that it could bind FGF receptors, induced the mitogen-associated protein kinase pathway but it induced neither cell proliferation nor neuronal differentiation.^11, 15, 25^ However, the activity of the intracellular FGF1^K132E^ remained unknown.

We also studied two other mutants of the C-terminal domain of FGF1. Mutation of the serine 130 into an alanine or aspartic acid was performed to examine the role of FGF1 phosphorylation on FGF1 intracrine activities. Indeed, FGF1 is phosphorylated on serine 130 by the protein kinase C delta (PKC*δ*) in the nucleus.^26, 27^ Mutation of this serine into an alanine abolishes FGF1 phosphorylation, while its mutation into an aspartic acid mimics FGF1 phosphorylation. Both recombinant FGF1 mutants could bind FGF receptors, be internalized in cells and translocated to the nucleus but they presented a reduced mitogenic activity.^28^ In the present study, FGF1^K132E^, FGF1^S130A^ and FGF1^S130D^ were overexpressed in PC12 cells to examine their intracellular activities. We showed that all these FGF1 mutants display a nuclear and cytosolic localization. However, their activities differed. The K132E mutation inhibited both neurotrophic and anti-apoptotic activities of FGF1. Both FGF1^S130A^ and FGF1^S130D^ mutants could induce PC12 cells neuronal differentiation, whereas only FGF1^S130A^ protected PC12 cells from p53-dependent apoptosis. Altogether our study shows that the FGF1 C-terminal domain is involved in the regulation of both its neurotrophic and its anti-apoptotic activity and that FGF1 phosphorylation regulates its anti-apoptotic activity whereas it does not interfere with its neuronal differentiation activity.

## Results

### FGF1^K132E^ expression and subcellular localization

**Figure 1:**
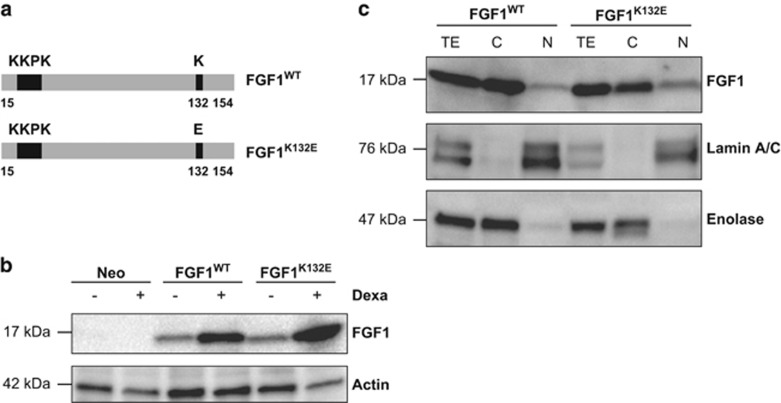
FGF1^K132E^ expression and subcellular localization in PC12 cells. (**a**) PC12 cells were transfected with pLK-FGF1^WT^ or pLK-FGF1^K132E^ dexamethasone-inducible vectors to overexpress FGF1^WT^ or FGF1^K132E^, respectively. The pLK-FGF1^K132E^ vector was generated by site-directed mutagenesis using pLK-FGF1^WT^ (aa 15 to 154) as a DNA template. The KKPK sequence (aa 23 to 27) is the FGF1 nuclear localization sequence. (**b**) Neo, FGF1^WT^, FGF1^K132E^ PC12 cell lines were cultured in the absence or presence of 5 × 10^−7^ M dexamethasone for 48 h. FGF1 expression was analyzed by western blot using the actin level as a control. The presence of dexamethasone increased FGF1 levels in FGF1^WT^ and FGF1^K132E^ PC12 cells, no FGF1 was detected in Neo PC12 cells. (**c**) FGF1^WT^ and FGF1^K132E^ PC12 cell lines were treated with dexamethasone for 48 h. Nuclear (N) and cytosolic (C) proteins were analyzed by western blot for FGF1, Enolase (cytosolic marker) and Lamin A/C (nuclear marker). Total protein extracts (TE) were used as controls. FGF1 was detected in all the fractions

To progress in the characterization of the intracellular FGF1 pathway, we first examined the cell fate and activity of the FGF1^K132E^ mutant in PC12 cells. We stably transfected PC12 cells with dexamethasone-inducible expression vectors encoding either wild-type (FGF1^WT^) or mutant (FGF1^K132E^) FGF1 (Figure 1a). PC12 cells transfected by the empty vector served as a control (Neo).

Stable Neo, FGF1^WT^ and FGF1^K132E^ PC12 cell lines were cultured in the absence or presence of dexamethasone for 48 h to induce FGF1 expression. FGF1 protein levels were analyzed by western blot (Figure 1b). Endogenous rat FGF1 was undetectable by western blot in control cells. In the absence of dexamethasone, FGF1 is detectable in FGF1^WT^ and FGF1^K132E^ PC12 cell lines, suggesting that the MMTV-LTR promoter is leaky in the absence of glucocorticoid addition. However, a threefold increase of FGF1 levels was detected in FGF1^WT^ and FGF1^K132E^ PC12 cells lines after dexamethasone treatment.

We previously showed that FGF1 nuclear localization is required for its intracellular activities in PC12 cells.^17^ To study FGF1^K132E^ subcellular localization, cytosolic and nuclear protein extracts from FGF1^WT^ and FGF1^K132E^ PC12 cell lines were analyzed by western blot (Figure 1c). Enolase and Lamin A/C were used as purity controls for cytosolic and nuclear fractions, respectively. Both proteins were mainly detected in their respective fraction. In transfected PC12 cells, FGF1^WT^ and FGF1^K132E^ were both detected in cytosolic and nuclear fractions suggesting that FGF1^K132E^ can be translocated to the nucleus, similarly as FGF1^WT^.

### The K132E mutation inhibits FGF1 neurotrophic activity

**Figure 2:**
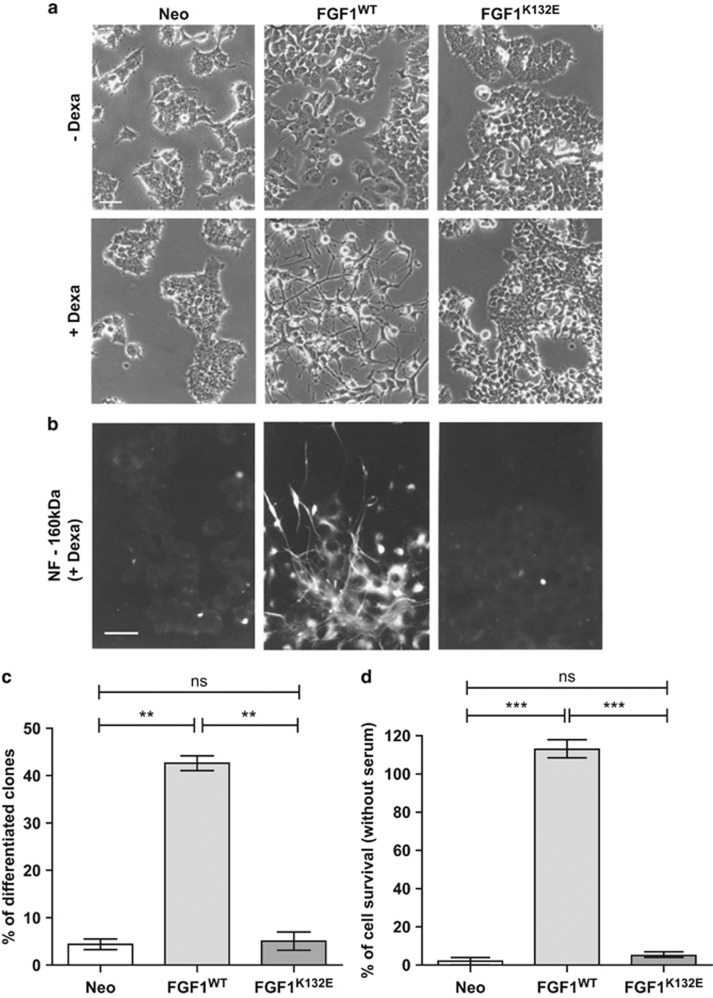
The K132E mutation inhibits FGF1 neurotrophic activity. (**a** and **b**) Neo, FGF1^WT^ and FGF1^K132E^ PC12 cell lines were cultured in the absence or presence of dexamethasone for 7 days. Cell morphology was observed by phase contrast microscopy (**a**) and Neurofilament NF-160 kDa (a neuronal marker) expression was analyzed by immunocytochemistry (**b**). Scale bars, 50 *μ*m (**a**); 25 *μ*m (**b**). (**c**) PC12 cells transfected by pLK-Neo, pLK-FGF1^WT^ and pLK-FGF1^K132E^ were treated with dexamethasone for 10 days in a selection medium. The clones morphology was examined and the percentage of differentiated clones, which present neuritis longer than cell size, were quantified from four independent experiments (**P<0.01). (**d**) Neo, FGF1^WT^ and FGF1^K132E^ PC12 cell lines were cultured in the presence of dexamethasone in serum-free medium for 11 days. Cell survival was analyzed by crystal violet nuclei staining. The graph presents the mean of three independent experiments (***P<0.001). In contrast to FGF1^K132E^, FGF1^WT^ presented a neurotrophic activity

In PC12 cells, intracellular FGF1 induced both neuronal differentiation and cell survival in serum-free medium.^11, 17^ To test if the K132E mutation could modify the intracellular FGF1 neurotrophic activity, PC12 cell lines (Neo, FGF1^WT^ and FGF1^K132E^) were cultured for 7 days in the absence or presence of dexamethasone to induce FGF1 expression. Cell morphology was then observed by phase contrast microscopy (Figure 2a). In the absence of dexamethasone, PC12 cells (Neo, FGF1^WT^ and FGF1^K132E^) presented an undifferentiated phenotype. In the presence of dexamethasone, only FGF1^WT^ PC12 cells presented long and ramified neuritis, which is characteristic of PC12 cells neuronal differentiation. We also analyzed the neurofilament NF-160 kDa expression by immunocytochemistry (Figure 2b). After dexamethasone treatment, this neuronal marker could only be detected in FGF1^WT^ PC12 cells. To quantify the neurotrophic activity of both FGF1 forms, the percentage of differentiated clones was determined in a large population of transfected PC12 clones in the presence of dexamethasone (Figure 2c). For each expression vector, four independent transfections were performed and the morphology of at least 100 G418-resistant transfected PC12 clones was analyzed. After 10 days of dexamethasone treatment, up to 40% of the FGF1^WT^ transfected PC12 clones presented a differentiated phenotype. However, only 4–5% of FGF1^K132E^ or Neo-transfected PC12 clones presented a differentiated phenotype in these conditions. Thus, in contrast to FGF1^WT^, intracellular FGF1^K132E^ does not induce PC12 cells differentiation.

To determine if the K132E mutation inhibits all aspects of FGF1 neurotrophic activity, cell survival in the absence of serum was examined in transfected PC12 cell lines (Neo, FGF1^WT^ and FGF1^K132E^). These cell lines were cultured in serum-free medium containing dexamethasone during 11 days (Figure 2d). Cell survival was quantified after crystal violet nuclei staining. Only FGF1^WT^ protected PC12 cells from serum-depletion-induced apoptosis. Most of FGF1^K132E^ PC12 cells died in the absence of serum, at a similar level to Neo PC12 cells. Thus, the K132E mutation inhibits the neurotrophic activity of intracellular FGF1 in PC12 cells.

### The K132E mutation inhibits the FGF1 anti-apoptotic activity during p53-dependent cell death

**Figure 3:**
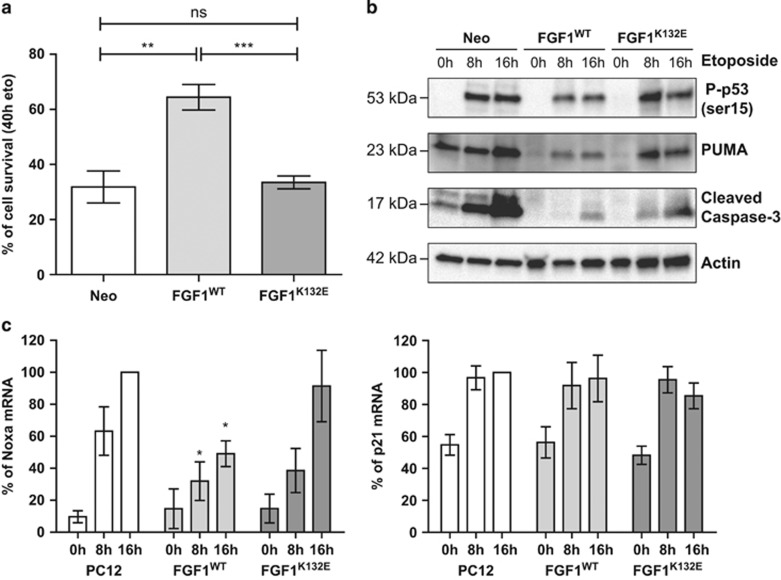
The K132E mutation inhibits FGF1 anti-apoptotic activity. (**a**) Neo, FGF1^WT^ and FGF1^K132E^ PC12 cell lines were treated with dexamethasone for 48 h. Then, cell survival after 40 h etoposide treatment was estimated after crystal violet nuclei staining. FGF1^WT^ protected PC12 cells from p53-dependent apoptosis whereas FGF1^K132E^ did not (**P<0.01, ***P<0.001, ns *P*>0.05, *n*=4). (**b**) Neo, FGF1^WT^ and FGF1^K132E^ PC12 cell lines cultured in the presence of dexamethasone were treated with etoposide for 0, 8 or 16 h. p53 activation (Ser 15 phosphorylation), PUMA expression and caspase-3 cleavage were analyzed by western blot. Actin detection was used as a control. Etoposide induced upregulation of P-p53 (Ser 15), PUMA and cleaved caspase-3 in all cells. However, these levels were lower in FGF1^WT^ PC12 cells compared with FGF1^K132E^ and Neo PC12 cells. (**c**) Noxa (left panel) and p21 (right panel) mRNA levels were analyzed by RT-PCR in native, FGF1^WT^ and FGF1^K132E^ PC12 cell lines after 0, 8 or 16 h of etoposide treatment in the presence of dexamethasone. The 18 S rRNA levels were used as a control for the quantifications. In contrast to FGF1^K132E^, FGF1^WT^ decreased p53-dependent up-regulation of *noxa* mRNA levels (*P<0.5, *n*=3)

We have previously shown that intracellular FGF1 protects PC12 cells from p53-dependent apoptosis.^17^ In this study, we tested the anti-apoptotic activity of the FGF1^K132E^ mutant in this cell death process. After 2 days of dexamethasone treatment, PC12 cell lines (Neo, FGF1^WT^ and FGF^K132E^) were treated with 50 *μ*g/ml etoposide to induce p53-dependent apoptosis. Cell survival after 40 h of etoposide treatment was quantified after crystal violet nuclei staining (Figure 3a). The percentages of cell survival for Neo and FGF1^K132E^ PC12 cells were of 31 and 33%, respectively, which is not significantly different. By contrast, FGF1^WT^ PC12 survival appeared to be significantly higher (64%) than FGF1^K132E^ and Neo PC12 cell survival.

We then analyzed different markers of p53-dependent apoptosis by western blot (Figure 3b). We examined the levels of serine 15-phosphorylated p53 (a marker of p53 activation), the levels of p53-upregulated modulator of apoptosis (PUMA; a pro-apoptotic BH3-only protein encoded by a p53-transcriptional-target gene) and the cleavage of caspase-3 (i.e., the activation of this caspase). After two days of dexamethasone treatment, PC12 cell lines (Neo, FGF1^WT^ and FGF1^K132E^) were treated with etoposide during 0, 8 and 16 h. Etoposide addition increased phospho-p53 (P-p53) (Ser15), PUMA and cleaved caspase-3 levels in all the tested cell lines. However, all three protein levels appeared lower in FGF1^WT^ PC12 cells compared with Neo and FGF1^K132E^ PC12 cells. We also analyzed the expression levels of the two other p53 target genes *noxa* and *p21* by RT-PCR (Figure 3c). Etoposide treatment increased *noxa* and *p21* mRNA levels in all the tested cell lines. However, this accumulation was lower in FGF1^WT^ PC12 cells than in native and FGF1^K132E^ PC12 cells for *noxa* mRNA, which codes for a pro-apoptotic BH3-only member of Bcl-2 family. No significant difference was detected for *p21* mRNAs in the different cell lines.

Thus, FGF1^WT^ protects PC12 cells from p53-dependent apoptosis in contrast to FGF1^K132E^. In the presence of etoposide, FGF1^WT^ decreased p53 activation, p53-dependent trans-activation of pro-apoptotic genes (*PUMA* and *noxa*) and caspase activation, which resulted in a decrease in cell death. The K132E mutation of FGF1 decreased all of these effects.

Altogether, our study of the FGF1^K132E^ mutant in PC12 cells showed that the K132E mutation of FGF1 inhibits both its neurotrophic and anti-apoptotic activities. However, it does not inhibit its nuclear translocation. Thus, the K132E mutation probably inhibits a nuclear event required for nuclear FGF1 activities that remains to be determined.

### FGF1 phosphorylation does not modify its subcellular localization in PC12 cells

**Figure 4:**
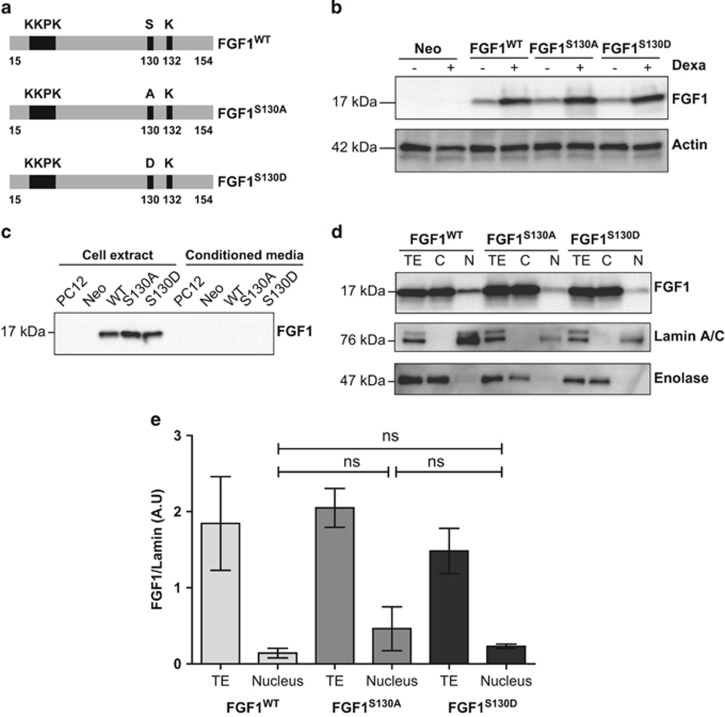
Expression and subcellular localization of wild-type and phosphorylation mutant forms of FGF1. (**a**) PC12 cells were transfected with the pLK-FGF1^WT^, pLK-FGF1^S130A^ or pLK-FGF1^S130D^ dexamethasone-inducible vectors to respectively overexpress FGF1^WT^, FGF1^S130A^ or FGF1^S130D^. The pLK-FGF1^S130A^ and pLK-FGF1^S130D^ vectors were generated by site-directed mutagenesis. (**b**) Neo, FGF1^WT^, FGF1^S130A^ and FGF1^S130D^ PC12 cell lines were cultured in the absence or presence of 5 × 10^−7^ M dexamethasone for 48 h. FGF1 expression was analyzed by western blot using actin level as a control. The presence of dexamethasone increased FGF1 levels at comparable levels in the different PC12 cells transfected to express one of the different FGF1 forms. (**c**) After heparin sepharose concentration, FGF1 levels in cell extracts and conditioned media of native, Neo, FGF1^WT^, FGF1^S130A^ and FGF1^S130D^ PC12 cells were examined. (**d**) FGF1^WT^, FGF1^S130A^ and FGF1^S130D^ PC12 cell lines were treated with dexamethasone for 48 h. Nuclear (N) and cytosolic (C) proteins were analyzed by western blot for FGF1, Enolase (cytosolic marker) and Lamin A/C (nuclear marker). Total protein extracts (TE) were used as controls. FGF1 was detected in all the fractions. (**e**) Quantification of the levels of FGF1 normalized to Lamin A/C levels in the total lysates and nuclear fractions of three independent experiments

FGF1 can be phosphorylated on serine 130 by PKC*δ* in the nucleus.^15, 27^ To determine if FGF1 phosphorylation is involved in the regulation of FGF1 intracellular activities, PC12 cells were stably transfected with FGF1 phosphorylation mutant (FGF1^S130A^ or FGF1^S130D^) encoding dexamethasone-inducible expression vectors (Figure 4a). The S130A mutation prevents FGF1 phosphorylation whereas the S130D mutation mimics constitutive phosphorylation.

First, FGF1 protein levels were analyzed in PC12 cell lines (Neo, FGF1^WT^, FGF1^S130A^ and FGF1^S130D^). These cell lines were cultured in the absence or presence of dexamethasone for 48 h to induce FGF1 expression, and FGF1 levels were analyzed by western blot (Figure 4b). In control PC12 cells, the level of endogenous FGF1 was undetectable. In the three other PC12 cell lines (FGF1^WT^, FGF1^S130A^ and FGF1^S130D^), the level of FGF1 was low in the absence of dexamethasone and increased in its presence. FGF1^WT^, FGF1^S130A^ and FGF1^S130D^ PC12 cell lines expressed similar levels of FGF1 in the presence of dexamethasone.

After concentration on heparin sepharose, FGF1 was detected in cell extracts of the different cell lines but not in the conditioned media, thus showing that FGF1 is not secreted in these cells (Figure 4c). FGF1 subcellular localization was then examined in the different PC12 cell lines to determine if phosphorylation of FGF1 could modify the protein localization. Cytosolic and nuclear proteins extracted from PC12 cell lines (FGF1^WT^, FGF1^S130A^ and FGF1^S130D^) cultured in the presence of dexamethasone for 48 h were analyzed by western blot (Figure 4d). Total cell lysates were used as positive controls. Lamin A/C and Enolase detection were used as nuclear and cytosolic purity controls, respectively. FGF1 is detected in both nuclear and cytosolic fractions of all FGF1 expressing cell lines. No significant difference in the levels of nuclear FGF1 was detected between these cell lines (Figure 4e), which suggests that FGF1 phosphorylation does not modify FGF1 nuclear localization in PC12 cells.

### Wild-type and phosphorylation mutant forms of FGF1 induce PC12 cell neuronal differentiation

**Figure 5:**
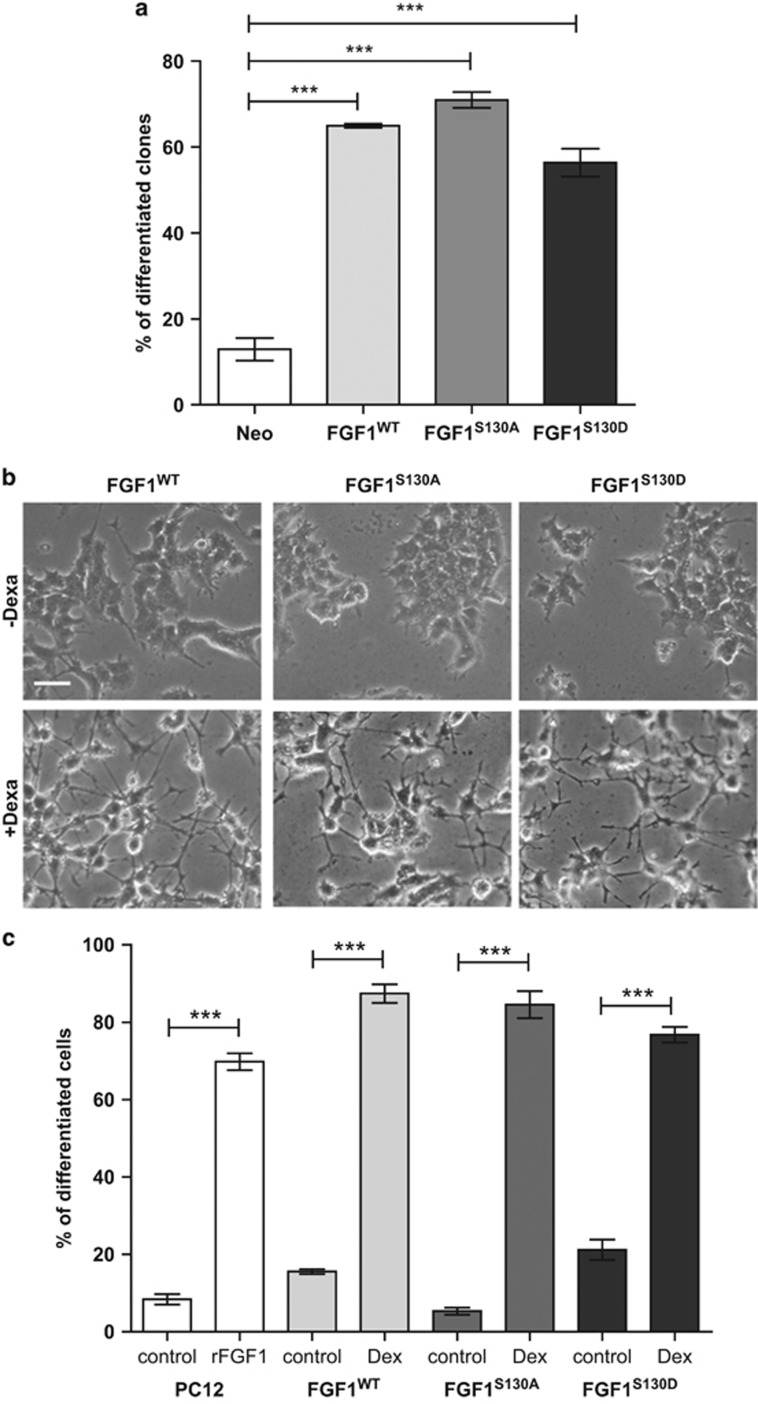
FGF1 phosphorylation does not modify its differentiation activity. (**a**) PC12 cells transfected with pLK-Neo, pLK-FGF1^WT^, pLK-FGF1^S130A^ or pLK-FGF1^S130D^ were treated with dexamethasone for 12 days in a selection medium. The morphology of the clones was examined and the percentages of differentiated clones, which present neuritis longer than cell size, were quantified from three independent experiments (***P<0.001). (**b**) FGF1^WT^, FGF1^S130A^ and FGF1^S130D^ PC12 cell lines were cultured in the absence or presence of dexamethasone for 3 days and the cell morphology was observed by phase contrast microscopy. Scale bar, 50 *μ*m. (**c**) Quantification of differentiation in FGF1^WT^, FGF1^S130A^ and FGF1^S130D^ PC12 cell lines in the absence or presence of dexamethasone. The differentiation of PC12 cells treated with 100 ng/ml recombinant FGF1 and 10 *μ*g/ml heparin (noted rFGF1) was evaluated as a control. FGF1^WT^, FGF1^S130A^ and FGF1^S130D^ induced PC12 cell differentiation

To determine the differentiation activity of FGF1 phosphorylation mutant forms, the percentage of differentiated clones was determined from a large population of transfected PC12 clones, as described above (Figure 5a). After 12 days of dexamethasone treatment, about 60% of the FGF1^WT^-, FGF1^S130A^- and FGF1^S130D^-transfected PC12 clones presented a differentiated phenotype, suggesting that FGF1 phosphorylation mutants could induce PC12 cell differentiation as well as wild-type FGF1.

To confirm this result, PC12 cell lines expressing FGF1^WT^, FGF1^S130A^ or FGF1^S130D^ were cultured in the absence or presence of dexamethasone for 3 days and cell morphology was observed by phase contrast microscopy (Figures 5b and c). In the absence of dexamethasone, FGF1^WT^-, FGF1^S130A^- and FGF1^S130D^-expressing PC12 cell lines presented an undifferentiated phenotype. By contrast, all these cell lines presented long and ramified neuritis after dexamethasone treatment. Overexpression of FGF1^WT^, FGF1^S130A^ or FGF1^S130D^ induced PC12 cell differentiation at a level which was comparable to the differentiation induced by the addition of recombinant FGF1 (rFGF1) in the culture medium. However, the addition of either an inhibitor of FGF receptors (PD173074) or a neutralizing antibody targeting FGF1 (AF232) in the culture medium strongly decreased rFGF1-induced PC12 cell differentiation, but had no effect on FGF1^WT^, FGF1^S130A^ and FGF1^S130D^ PC12 cell differentiation, confirming an intracellular mode of action of FGF1 and its mutants in our PC12 cell lines (Supplementary Figures 1–4). Altogether, our experiments show that the mutation of serine 130 to an alanine or aspartic acid does not modify the differentiation activity of intracellular FGF1 in PC12 cells, indicating that FGF1 phosphorylation does not regulate this activity.

### FGF1 phosphorylation inhibits its anti-apoptotic activity in p53-dependent cell death

**Figure 6:**
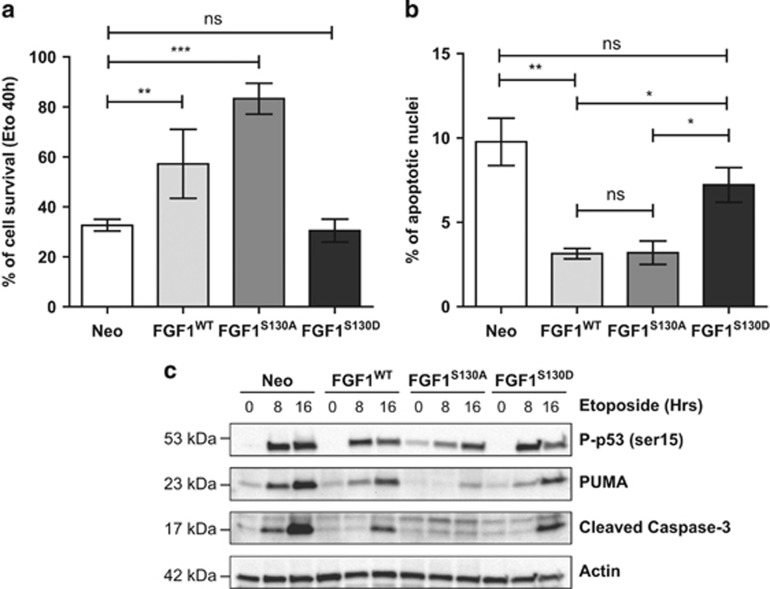
FGF1 phosphorylation inhibits its anti-apoptotic activity. (**a** and **b**) Neo, FGF1^WT^, FGF1^S130A^ and FGF1^S130D^ PC12 cell lines were treated with dexamethasone for 48 h. p53-dependent apoptosis was then induced by etoposide treatment during 40 h. (**a**) Cell survival was estimated by crystal violet nuclei staining (**P<0.01, ***P<0.001, ns *P*>0.05, *n*=6). (**b**) Apoptosis (percentage of apoptotic nuclei) was estimated after Hoechst nuclei staining (*P<0.05, **P<0.01, ns *P*>0.05, *n*=3). FGF1^WT^ and FGF1^S130A^ protected PC12 cells from p53-dependent apoptosis in contrast to FGF1^S130D^. (**c**) Neo, FGF1^WT^, FGF1^S130A^ and FGF1^S130D^ PC12 cells cultured in the presence of dexamethasone were treated with etoposide for 0, 8 or 16 h. The levels of *P*-p53 (Ser 15), PUMA and cleaved caspase-3 were detected by western blot. Actin was used as a loading control. FGF1^WT^, FGF1^S130A^ and FGF1^S130D^ decreased etoposide-induced upregulation of these different apoptotic markers. The stronger effect was observed for the unphosphorylable FGF1

We previously showed that intracellular FGF1^WT^ protects PC12 cells from p53-dependent apoptosis and that both ΔNLS and K132E FGF1 mutations inhibit this anti-apoptotic activity (Rodriguez-Enfedaque *et al.*^17^ and the present study). To determine if phosphorylation modulates FGF1 anti-apoptotic activity, PC12 cells (Neo, FGF1^WT^, FGF1^S130A^ and FGF1^S130D^) were cultured in the presence of dexamethasone for 48 h before the addition of etoposide to induce p53-dependent apoptosis. Cell survival after 40 h of etoposide treatment was analyzed after crystal violet nuclei staining (Figure 6a). As previously shown, about 60% of FGF1^WT^ PC12 cells survived, whereas this percentage fell to 30% for Neo PC12 cells. Expression of the non-phosphorylable FGF1^S130A^ highly protected PC12 cells against etoposide-induced apoptosis. Indeed, up to 80% of FGF1^S130A^ PC12 cells survived in these conditions. By contrast, expression of the phosphomimetic form FGF1^S130D^ did not protect PC12 cells from cell death, as similar levels of survival were observed with both Neo and FGF1^S130D^ PC12 cells. To confirm this result, we quantified the percentage of condensed and fragmented apoptotic nuclei after Hoechst staining, which characterizes late-stage apoptosis, on the different etoposide-treated PC12 cells (Figure 6b). The percentage of apoptotic nuclei was similar in FGF1^WT^ and FGF1^S130A^ PC12 cell lines. It was significantly reduced in these cells when compared with Neo and FGF1^S130D^ PC12 cells. Altogether, these different experiments show that FGF1^S130A^ protects PC12 cells from etoposide-induced apoptosis, which contrasts from FGF1^S130D^.

We then examined p53 phosphorylation, p53-dependent trans-activation of *PUMA* and capase-3 cleavage in Neo, FGF1^WT^, FGF1^S130A^ and FGF1^S130D^ PC12 cell lines after 0, 8 or 16 h of etoposide treatment (Figure 6c). In Neo PC12 cells, the levels of P-p53 serine 15 (Ser 15), PUMA and cleaved caspase-3 increased after etoposide treatment in a time-dependent manner. These increases were reduced in FGF1-expressing PC12 cells compared with Neo PC12 cells. The stronger effect on these apoptotic markers was observed for the unphosphorylable FGF1^S130A^. Interestingly, FGF1^S130D^ had a slight effect on these different makers, but remained unable to protect PC12 cells from p53-dependent apoptosis. Altogether, our study showed that the unphosphorylable FGF1^S130A^ protects PC12 cells from p53-dependent apoptosis by acting efficiently at different levels of this cell death process, in contrast to the phosphomimetic FGF1^S130D^. Therefore, it appears that phosphorylation inhibits FGF1 anti-apoptotic activity.

## Discussion

**Figure 7:**
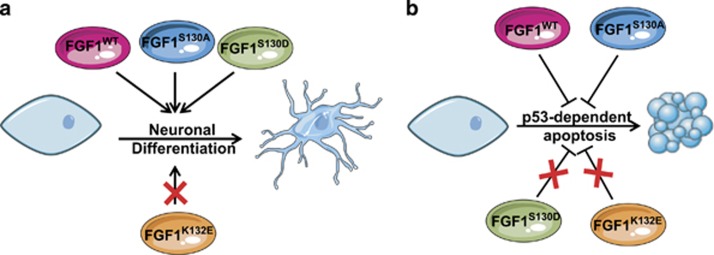
Intracellular activities and subcellular localization of wild-type (FGF1^WT^) and mutant FGF1 forms (FGF1^K132E^, FGF1^S130A^, FGF1^S130D^). (**a**) Intracellular FGF1^WT^ induced PC12 cells neuronal differentiation. Only the K132E mutation inhibited this activity. (**b**) Intracellular FGF1^WT^ and FGF1^S130A^ protected PC12 cells from p53-dependent apoptosis, in contrast to FGF1^K132E^ and FGF1^S130D^. All these FGF1 forms presented both a nuclear and a cytosolic localization

We have previously shown that FGF1 mediates neurotrophic and anti-apoptotic activities in PC12 cells by an intracrine pathway that requires FGF1 nuclear translocation.^11, 17^ In the present study, we examined the activities of FGF1 mutant forms affected in their C-terminus (FGF1^K132E^, FGF1^S130A^ and FGF1^S130D^; Figure 7). We showed that all these mutants displayed a nuclear and cytosolic localization but differed in their activities. Indeed, the K132E mutation inhibited FGF1 neurotrophic and anti-apoptotic activities, whereas changing serine 130 into an alanine or aspartic acid did not alter FGF1-induced differentiation. Interestingly, the S130D mutation decreased the inhibitory activity of FGF1 on p53-dependent cell death, whereas the S130A mutation seemed to reinforce this activity. Altogether, our data show that the C-terminal domain of FGF1 is crucial for FGF1 intracellular activities. FGF1 nuclear translocation was not affected, suggesting that the observed effects are not related to differences in nucleo-cytoplasmic distribution of the mutated forms of FGF1. Furthermore, as FGF1 nuclear translocation is required for its neurotrophic and anti-apoptotic activities,^17^ these results imply that nuclear events, which are mitigated by the serine 130 and/or lysine 132 mutations, are required for FGF1 intracellular activities. These nuclear events could be FGF1 post-translational modifications and/or protein–protein interactions with regulators of cell differentiation and survival, such as transcriptional factors.

FGF1 had previously been shown to be post-translationally modified by methylation and phosphorylation. Indeed, FGF1 can be methylated on 3 of its 12 lysine residues.^29^ *In vitro* reduction of bovine-purified FGF1 methylation reduced both FGF1 affinity for heparin and its mitogenic activity when added to the culture medium of Balb/C 3T3 cells. The lysine 132 that is mutated in our study is one of the lysines that could be methylated (referred as Lysine 118 in Harper and Lobb^29^). The recombinant FGF1^K132E^ mutant was studied by different laboratories.^11, 25^ This mutation was shown to inhibit FGF1 interaction with heparin and both FGF1 mitogenic and neurotrophic activities. However, recombinant FGF1^K132E^ could interact with FGF receptors and activate the mitogen-associated protein kinase pathway, just as recombinant wild-type FGF1.^11, 25^ This suggests that the absence of activity of this mutant does not rely on an inhibition of its interaction with heparin. Moreover, recombinant FGF1^K132E^ can be internalized by cells and translocated in the nucleus, like recombinant FGF1^WT^, but it fails to induce DNA synthesis in various cell types.^15^ We show here that intracellular FGF1^K132E^ can translocate to the nuclear compartment like wild-type FGF1, but fails to induce PC12 cell differentiation and survival after serum depletion or p53-dependent apoptosis. Klingenberg *et al.*^15^ proposed that mutation of lysine 132 could interfere with FGF1 phosphorylation. Our study of the phosphorylation mutant forms of FGF1 (FGF1^S130A^ and FGF1^S130A^) in PC12 cells, which presented different activities from FGF1^K132E^ does not support this hypothesis. Thus, we favor the hypothesis that this mutation modifies the FGF1 ternary structure or interactions between FGF1 and nuclear proteins required for its activity. These modifications remain to be characterized.

FGF1 was previously shown to be phosphorylated on its serine 130 by PKC*δ* in the nucleus.^26, 27^ The effect of adding recombinant FGF1^S130A^ and FGF1^S130E^ in the culture medium was previously analyzed by Wiedlocha's team.^27, 28^ Both recombinant FGF1 mutants were shown to bind FGF receptors, internalize and translocate in the nucleus, as well as to promote DNA synthesis, like recombinant FGF1^WT^. However, the phosphomimetic FGF1^S130E^ was shown to be exported to the cytosol after nuclear translocation, to be degraded. In contrast, FGF1^S130A^ remains in the nucleus, suggesting that FGF1 phosphorylation is involved in this cytosolic export process.^27^ We showed that FGF1^WT^, FGF1^S130A^ and FGF1^S130D^ could be translocated in the nucleus of PC12 cells. Quantification of nuclear FGF1 levels in our cell lines did not reveal any significant difference, suggesting that FGF1 phosphorylation did not interfere with FGF1 nuclear localization in PC12 cells. Different hypotheses could explain the discrepancies between both studies. Different cell types and different mutations were used to mimic FGF1 phosphorylation; another possible explanation is that recombinant internalized FGF1 and intracellular FGF1 display different regulations and fates in the cell.

The results reported here strongly suggest that phosphorylation inhibits FGF1 protection against p53-dependent apoptosis. A similar result has also been obtained using the human neuroblastoma SHSY-5Y cell line (data not shown). As FGF1 is a survival factor for a large range of cell types^11, 12, 24^ and is overexpressed in various tumors,^19, 30, 31, 32^ it would be interesting to characterize the nuclear events involved in the FGF1 anti-apoptotic activity (i.e., nuclear protein interactions and/or transcriptional regulations) that are affected by FGF1 phosphorylation. Other growth factors (FGF2 and FGF3) and growth factor receptors (FGFR1 and EGFR (epidermal growth factor receptor)) could also mediate their activity by an intracrine/nuclear pathway.^5, 6, 33, 34, 35, 36^ As most of these nuclear growth factors and/or receptors could exert oncogenic activities, the study of these nuclear pathways could be crucial to identify key regulators involved in tumor progression and/or resistance to radio- and/or chemo-therapy.

## Materials and Methods

### Cell culture and drugs

PC12 cells, a rat pheochromocytoma-derived cell line^37^ (originally obtained from P. Brachet, Angers), were cultured in Dulbecco's modified Eagle's medium (DMEM) supplemented with 10% fetal calf serum (FCS), 5% horse serum (HS), 100 *μ*g/ml penicillin, 100 U/ml streptomycin and 1% Glutamax at 37 °C in a humidified atmosphere of 5% CO_2_ as previously described.^17^ PC12 cells transfected with inducible FGF1 expression vectors (pLK-Neo, pLK-FGF1^WT^, plK-FGF1^K132E^, pLK-FGF1^S130A^ and pLK-FGF1^S130D^) were maintained in DMEM supplemented with serum depleted in glucocorticoid as previously described.^17^ FGF1 expression in transfected cells was induced by 5 × 10^−7^ M dexamethasone (Tebu). Etoposide (50 *μ*g/ml, Sigma-Aldrich, St Louis, MO, USA, E1383) was used to induce p53-dependent apoptosis.

### PC12 cells transfection

Dexamethasone-inducible mutant FGF1 expression vectors were generated using QuickChange II XL Site-Directed Mutagenesis Kit (Agilent Technologies, Santa Clara, CA, USA) according to the protocol of the manufacturer. We used the pLK-FGF1^WT^ vector as a DNA template to generate pLK-FGF1^K132E^, pLK-FGF1^S130A^ and pLK-FGF1^S130D^ with specific primers.

PC12 cells were transfected with 10 *μ*g of the different vectors and 60 *μ*l of Lipofectin reagent (Life Technologies, Carlsbad, CA, USA) in 100 mm diameter Petri dishes as previously described.^11^ Two days after transfection, cells were trypsinized and replated in four 100 mm diameter Petri dishes with a selection medium, which contains 0.5 mg/ml G418 (Life Technologies) in a glucocorticoid-depleted culture medium. After 15 days of selection, geneticin resistant colonies appeared whatever the expression vector used. WT and mutant FGF1-transfected PC12 cell lines were isolated and amplified for further analysis.

### PC12 cells neuronal differentiation

For each transfection, cells were treated with 5 × 10^−7^ M dexamethasone in a selection medium to induce FGF1 expression. After 10–12 days of treatment, the cell morphology was observed by phase contrast microscopy. The number of non-differentiated clones (composed of cells with no neuritis extension or extensions smaller than the size of the soma) and differentiated clones (composed of cells extending neuritis longer than the size of the cell body) were quantified. For each expression vector, four independent experiments were performed and at least 100 clones were examined by plate.

PC12 cell neuronal differentiation was also examined in stable PC12 cell lines after 3–7 days of 5 × 10^−7^ M dexamethasone treatment by phase-contrast microscopy using a Nikon TMS microscope. Pictures were taken with a Nikon D50 camera and the percentage of differentiated PC12 cells was determined. Differentiation of PC12 cells after addition of 100 ng/ml of recombinant FGF1 (R&D Systems, Minneapolis, MN, USA) and 10 *μ*g/ml of Heparin in the culture media was performed as a control. In some of the experimental conditions, 10–100 nM of PD173074 (R&D systems) were added to inhibit FGF receptors and test the differentiation of PC12 cells after overexpressing FGF1 or treating with recombinant FGF1. We also tested the effect of an anti-FGF1 antibody (AF232, R&D Systems) added at a concentration of 12.5 and 25 *μ*g/ml in the culture media to measure its neutralizing potency on the differentiation activity of recombinant FGF1 (25 and 50 ng/ml) or overexpressed FGF1 in PC12 cells. We also examined the expression of the 160 kDa form of Neurofilament (NF-160, N5264, Sigma-Aldrich) by immunocytochemistry as a marker of neuronal differentiation (previously described in Rodriguez-Enfedaque *et al.*^17^).

### Nucleo-cytoplasmic fractionation

Cell fractionation was performed with NE-PER Nuclear and Cytoplasmic Extraction Reagents kit (Thermo Scientific). About 10^7^ cells were used, according to the protocol of the manufacturer. Fractions were then analyzed by western blot to detect FGF1, Enolase and Lamin A/C in the different fractions.

### Cell survival analysis by crystal violet nuclei staining

PC12 cell lines were plated in 12-multiwell plates with 5 × 10^−7^ M dexamethasone. When the cells reached 70% confluence, 50 *μ*g/ml etoposide was added to the medium to induce p53-dependent apoptosis (as previously described Bouleau *et al.*^16^). Cell viability was estimated using the crystal violet method (0.1% crystal violet, 0.1 M citric acid) after 40 h of etoposide treatment.

Cell survival in the absence of serum was also analyzed by crystal violet nuclei staining. PC12 cell lines were plated in the presence of dexamethasone in low-serum medium (1% FCS and 0.5% HS) to permit cell attachment. Four days later, the cells were cultured for further 11 days in serum-free medium. Percentages of cell survival were determined after crystal violet nuclei staining as described above.

### Apoptosis analysis by hoechst staining assay

Morphological changes in the nuclear chromatin of cells undergoing apoptosis were detected by Hoechst 33342 staining. WT and mutant FGF1-transfected PC12 cells were grown on glass coverslips in 6-well plates. After 48 h of dexamethasone treatment, 50 *μ*g/ml etoposide was added to the medium to induce p53-dependent apoptosis during 40 h. Cells were then fixed with 3.7% PBS-formaldehyde for 20 min at room temperature. Fixed cells were washed with PBS and incubated with 10 *μ*g/ml Hoechst for 5 min and analyzed by epifluorescence microscopy. Pictures were taken on a DMR Leica microscope equipped with an Olympus DP70 photo camera. For each cell line, about 1000 nuclei were analyzed to quantify the number of condensed and/or fragmented nuclei (late marker of apoptotic cells) reported to the total number of nuclei.

### Western blot analysis

PC12 cell lines were plated in 60 mm dishes in the absence or presence of 5 × 10^−7^ M dexamethasone. After 48 h of dexamethasone treatment, cells were incubated with 50 *μ*g/ml etoposide to induce p53-dependent apoptosis. After different etoposide treatment times (0, 4, 8 or 16 h), cells were harvested, lysed and frozen at −20 °C. Proteins (10–20 *μ*g) were analyzed by western blot (as previously described^17^). The primary antibodies used in this study were anti-FGF1 (AB-32-NA, R&D Systems), anti-p53-P (Ser-15) (9284, Cell Signaling, Danvers, MA, USA), anti-PUMA*α* (N-19, Santa Cruz, Dallas, TX, USA), anti-cleaved Caspase-3 (Asp175, Cell Signaling), anti-actin (A2066, Sigma-Aldrich), anti-Lamin A/C (2032, Cell Signaling) and anti-Enolase (C-19, Santa Cruz).

To test the presence of FGF1^WT^, FGF1^S130A^ and FGF1^S130D^ in the conditioned media, FGF1 heparin sepharose concentration was performed. Briefly, 120 *μ*g of total cell extract proteins or the equivalent fraction of conditioned media (usually about 1 ml) from dexamethasone-treated PC12 cell lines was incubated with 150 *μ*l of heparin sepharose (CL-6B, GE Healthcare, Little Chalfont, UK) in PBS containing 0.66 M NaCl and protease inhibitors (dilution 1/100, Roche, Basel, Switzerland). After one night of fixation at 4 °C, the heparin sepharose was washed three times with the binding buffer, and the heparin-binding proteins were then eluted in 60 *μ*l NuPage LDS sample buffer 2 × (Life Technologies) containing 100 mM DTT at 96 °C for 10 min.

### *noxa* and *p21* mRNA analysis by RT-PCR assay

PC12 cell lines were plated in 100-mm dishes in the presence of 5 × 10^−7^ M dexamethasone. After 48 h of dexamethasone treatment, cells were incubated with 50 *μ*g/ml etoposide to induce p53-dependent apoptosis. After different etoposide treatment times (0, 8 or 16 h), total RNAs were extracted using the guanidium isothiocyanate method. RT-PCR was performed to examine the levels of *noxa* and *p21* mRNA as previously described.^38^ The quantifications of *noxa* and *p21* mRNA were normalized with respect to 18S rRNA levels.

### Statistical analysis

Each bar of the different graphs indicates the average measure and standard deviation of the mean of at least three independent experiments, and *P*-values are from paired two-tailed Student's *t*-tests.

## Abbreviations

DMEM: Dulbecco's modified Eagle's medium
FCS: fetal calf serum
FGF: fibroblast growth factor
FGF1: fibroblast growth factor 1
FGFR: fibroblast growth factor receptor
HS: horse serum
NLS: nuclear localization sequence
PKC *δ*: protein kinase C delta
P-p53: phospho-p53
PUMA: p53-upregulated modulator of apoptosis
Ser 15: Serine 15

## References

[bib1] Itoh N, Ornitz DM. Fibroblast growth factors: from molecular evolution to roles in development, metabolism and disease. J Biochem 2011; 149: 121–130. 20940169 10.1093/jb/mvq121 PMC3106964

[bib2] Powers CJ, McLeskey SW, Wellstein A. Fibroblast growth factors, their receptors and signaling. Endocr Relat Cancer 2000; 7: 165–197. 11021964 10.1677/erc.0.0070165

[bib3] Dorey K, Amaya E. FGF signalling: diverse roles during early vertebrate embryogenesis. Development 2010; 137: 3731–3742. 20978071 10.1242/dev.037689 PMC3747497

[bib4] Imamura T, Engleka K, Zhan X, Tokita Y, Forough R, Roeder D et al. Recovery of mitogenic activity of a growth factor mutant with a nuclear translocation sequence. Science 1990; 249: 1567–1570. 1699274 10.1126/science.1699274

[bib5] Sorensen V, Nilsen T, Wiedlocha A. Functional diversity of FGF-2 isoforms by intracellular sorting. Bioessays 2006; 28: 504–514. 16615083 10.1002/bies.20405

[bib6] Antoine M, Reimers K, Dickson C, Kiefer P. Fibroblast growth factor 3, a protein with dual subcellular localization, is targeted to the nucleus and nucleolus by the concerted action of two nuclear localization signals and a nucleolar retention signal. J Biol Chem 1997; 272: 29475–29481. 9368007 10.1074/jbc.272.47.29475

[bib7] Goldfarb M. Fibroblast growth factor homologous factors: evolution, structure, and function. Cytokine Growth Factor Rev 2005; 16: 215–220. 15863036 10.1016/j.cytogfr.2005.02.002 PMC3212846

[bib8] Jaye M, Howk R, Burgess W, Ricca GA, Chiu IM, Ravera MW et al. Human endothelial cell growth factor: cloning, nucleotide sequence, and chromosome localization. Science 1986; 233: 541–545. 3523756 10.1126/science.3523756

[bib9] Walicke PA. Basic and acidic fibroblast growth factors have trophic effects on neurons from multiple CNS regions. J Neurosci 1988; 8: 2618–2627. 3249247 10.1523/JNEUROSCI.08-07-02618.1988 PMC6569507

[bib10] Cuevas P, Carceller F, Gimenez-Gallego G. Acidic fibroblast growth factor prevents post-axotomy neuronal death of the newborn rat facial nerve. Neurosci Lett 1995; 197: 183–186. 8552294 10.1016/0304-3940(95)11926-n

[bib11] Renaud F, Desset S, Oliver L, Gimenez-Gallego G, Van Obberghen E, Courtois Y et al. The neurotrophic activity of fibroblast growth factor 1 (FGF1) depends on endogenous FGF1 expression and is independent of the mitogen-activated protein kinase cascade pathway. J Biol Chem 1996; 271: 2801–2811. 8576258 10.1074/jbc.271.5.2801

[bib12] Desire L, Courtois Y, Jeanny JC. Suppression of fibroblast growth factors 1 and 2 by antisense oligonucleotides in embryonic chick retinal cells *in vitro* inhibits neuronal differentiation and survival. Exp Cell Res 1998; 241: 210–221. 9633530 10.1006/excr.1998.4048

[bib13] Raguenez G, Desire L, Lantrua V, Courtois Y. BCL-2 is upregulated in human SH-SY5Y neuroblastoma cells differentiated by overexpression of fibroblast growth factor 1. Biochem Biophys Res Commun 1999; 258: 745–751. 10329457 10.1006/bbrc.1999.0613

[bib14] Wiedlocha A, Falnes PO, Madshus IH, Sandvig K, Olsnes S. Dual mode of signal transduction by externally added acidic fibroblast growth factor. Cell 1994; 76: 1039–1051. 7511061 10.1016/0092-8674(94)90381-6

[bib15] Klingenberg O, Widlocha A, Rapak A, Munoz R, Falnes P, Olsnes S. Inability of the acidic fibroblast growth factor mutant K132E to stimulate DNA synthesis after translocation into cells. J Biol Chem 1998; 273: 11164–11172. 9556604 10.1074/jbc.273.18.11164

[bib16] Bouleau S, Grimal H, Rincheval V, Godefroy N, Mignotte B, Vayssiere JL et al. FGF1 inhibits p53-dependent apoptosis and cell cycle arrest via an intracrine pathway. Oncogene 2005; 24: 7839–7849. 16091747 10.1038/sj.onc.1208932

[bib17] Rodriguez-Enfedaque A, Bouleau S, Laurent M, Courtois Y, Mignotte B, Vayssiere JL et al. FGF1 nuclear translocation is required for both its neurotrophic activity and its p53-dependent apoptosis protection. Biochim Biophys Acta 2009; 1793: 1719–1727. 19765618 10.1016/j.bbamcr.2009.09.010

[bib18] Dorkin TJ, Robinson MC, Marsh C, Neal DE, Leung HY. aFGF immunoreactivity in prostate cancer and its co-localization with bFGF and FGF8. J Pathol 1999; 189: 564–569. 10629559 10.1002/(SICI)1096-9896(199912)189:4<564::AID-PATH480>3.0.CO;2-1

[bib19] Smith G, Ng MT, Shepherd L, Herrington CS, Gourley C, Ferguson MJ et al. Individuality in FGF1 expression significantly influences platinum resistance and progression-free survival in ovarian cancer. Br J Cancer 2012; 107: 1327–1336. 22990650 10.1038/bjc.2012.410 PMC3494420

[bib20] Slattery ML, John EM, Stern MC, Herrick J, Lundgreen A, Giuliano AR et al. Associations with growth factor genes (FGF1, FGF2, PDGFB, FGFR2, NRG2, EGF, ERBB2) with breast cancer risk and survival: the Breast Cancer Health Disparities Study. Breast Cancer Res Treat 2013; 140: 587–601. 23912956 10.1007/s10549-013-2644-5 PMC3860319

[bib21] Eckenstein F, Woodward WR, Nishi R. Differential localization and possible functions of aFGF and bFGF in the central and peripheral nervous systems. Ann N Y Acad Sci 1991; 638: 348–360. 1723856 10.1111/j.1749-6632.1991.tb49045.x

[bib22] Stock A, Kuzis K, Woodward WR, Nishi R, Eckenstein FP. Localization of acidic fibroblast growth factor in specific subcortical neuronal populations. J Neurosci 1992; 12: 4688–4700. 1281493 10.1523/JNEUROSCI.12-12-04688.1992 PMC6575752

[bib23] Bugra K, Oliver L, Jacquemin E, Laurent M, Courtois Y, Hicks D. Acidic fibroblast growth factor is expressed abundantly by photoreceptors within the developing and mature rat retina. Eur J Neurosci 1993; 5: 1586–1595. 7510204 10.1111/j.1460-9568.1993.tb00228.x

[bib24] Renaud F, Oliver L, Desset S, Tassin J, Romquin N, Courtois Y et al. Up-regulation of aFGF expression in quiescent cells is related to cell survival. J Cell Physiol 1994; 158: 435–443. 7510293 10.1002/jcp.1041580307

[bib25] Burgess WH, Shaheen AM, Ravera M, Jaye M, Donohue PJ, Winkles JA. Possible dissociation of the heparin-binding and mitogenic activities of heparin-binding (acidic fibroblast) growth factor-1 from its receptor-binding activities by site-directed mutagenesis of a single lysine residue. J Cell Biol 1990; 111: 2129–2138. 1699952 10.1083/jcb.111.5.2129 PMC2116333

[bib26] Mascarelli F, Raulais D, Courtois Y. Fibroblast growth factor phosphorylation and receptors in rod outer segments. EMBO J 1989; 8: 2265–2273. 2551678 10.1002/j.1460-2075.1989.tb08351.x PMC401157

[bib27] Wiedlocha A, Nilsen T, Wesche J, Sorensen V, Malecki J, Marcinkowska E et al. Phosphorylation-regulated nucleocytoplasmic trafficking of internalized fibroblast growth factor-1. Mol Biol Cell 2005; 16: 794–810. 15574884 10.1091/mbc.E04-05-0389 PMC545912

[bib28] Klingenberg O, Wiedlocha A, Olsnes S. Effects of mutations of a phosphorylation site in an exposed loop in acidic fibroblast growth factor. J Biol Chem 1999; 274: 18081–18086. 10364261 10.1074/jbc.274.25.18081

[bib29] Harper JW, Lobb RR. Reductive methylation of lysine residues in acidic fibroblast growth factor: effect on mitogenic activity and heparin affinity. Biochemistry 1988; 27: 671–678. 3126814 10.1021/bi00402a027

[bib30] La Rosa S, Sessa F, Colombo L, Tibiletti MG, Furlan D, Capella C. Expression of acidic fibroblast growth factor (aFGF) and fibroblast growth factor receptor 4 (FGFR4) in breast fibroadenomas. J Clin Pathol 2001; 54: 37–41. 11271786 10.1136/jcp.54.1.37 PMC1731265

[bib31] Takahashi JA, Mori H, Fukumoto M, Igarashi K, Jaye M, Oda Y et al. Gene expression of fibroblast growth factors in human gliomas and meningiomas: demonstration of cellular source of basic fibroblast growth factor mRNA and peptide in tumor tissues. Proc Natl Acad Sci USA 1990; 87: 5710–5714. 2377607 10.1073/pnas.87.15.5710 PMC54397

[bib32] Maxwell M, Naber SP, Wolfe HJ, Hedley-Whyte ET, Galanopoulos T, Neville-Golden J et al. Expression of angiogenic growth factor genes in primary human astrocytomas may contribute to their growth and progression. Cancer Res 1991; 51: 1345–1351. 1705174

[bib33] Lo HW, Hung MC. Nuclear EGFR signalling network in cancers: linking EGFR pathway to cell cycle progression, nitric oxide pathway and patient survival. Br J Cancer 2006; 94: 184–188. 16434982 10.1038/sj.bjc.6602941 PMC2361115

[bib34] Stachowiak MK, Maher PA, Stachowiak EK. Integrative nuclear signaling in cell development—a role for FGF receptor-1. DNA Cell Biol 2007; 26: 811–826. 18021009 10.1089/dna.2007.0664

[bib35] Patry V, Bugler B, Maret A, Potier M, Prats H. Endogenous basic fibroblast growth factor isoforms involved in different intracellular protein complexes. Biochem J 1997; 326: 259–264. 9337877 10.1042/bj3260259 PMC1218663

[bib36] Payson RA, Chotani MA, Chiu IM. Regulation of a promoter of the fibroblast growth factor 1 gene in prostate and breast cancer cells. J Steroid Biochem Mol Biol 1998; 66: 93–103. 9719443 10.1016/s0960-0760(98)00051-x

[bib37] Greene LA, Tischler AS. Establishment of a noradrenergic clonal line of rat adrenal pheochromocytoma cells which respond to nerve growth factor. Proc Natl Acad Sci USA 1976; 73: 2424–2428. 1065897 10.1073/pnas.73.7.2424 PMC430592

[bib38] Bouleau S, Parvu-Ferecatu I, Rodriguez-Enfedaque A, Rincheval V, Grimal H, Mignotte B et al. Fibroblast growth factor 1 inhibits p53-dependent apoptosis in PC12 cells. Apoptosis 2007; 12: 1377–1387. 17473910 10.1007/s10495-007-0072-x

