# Supplementary material for: FGF1 C-terminal domain and phosphorylation regulate intracrine FGF1 signaling for its neurotrophic and anti-apoptotic activities
Source: Cell Death Dis. 2016 Feb 4;7(2):e2079–. doi: 10.1038/cddis.2016.2 (PMC4849156; doi:10.1038/cddis.2016.2)
Supplement: Supplementary Figures [file cddis20162x1.ppt]

## Slide 1
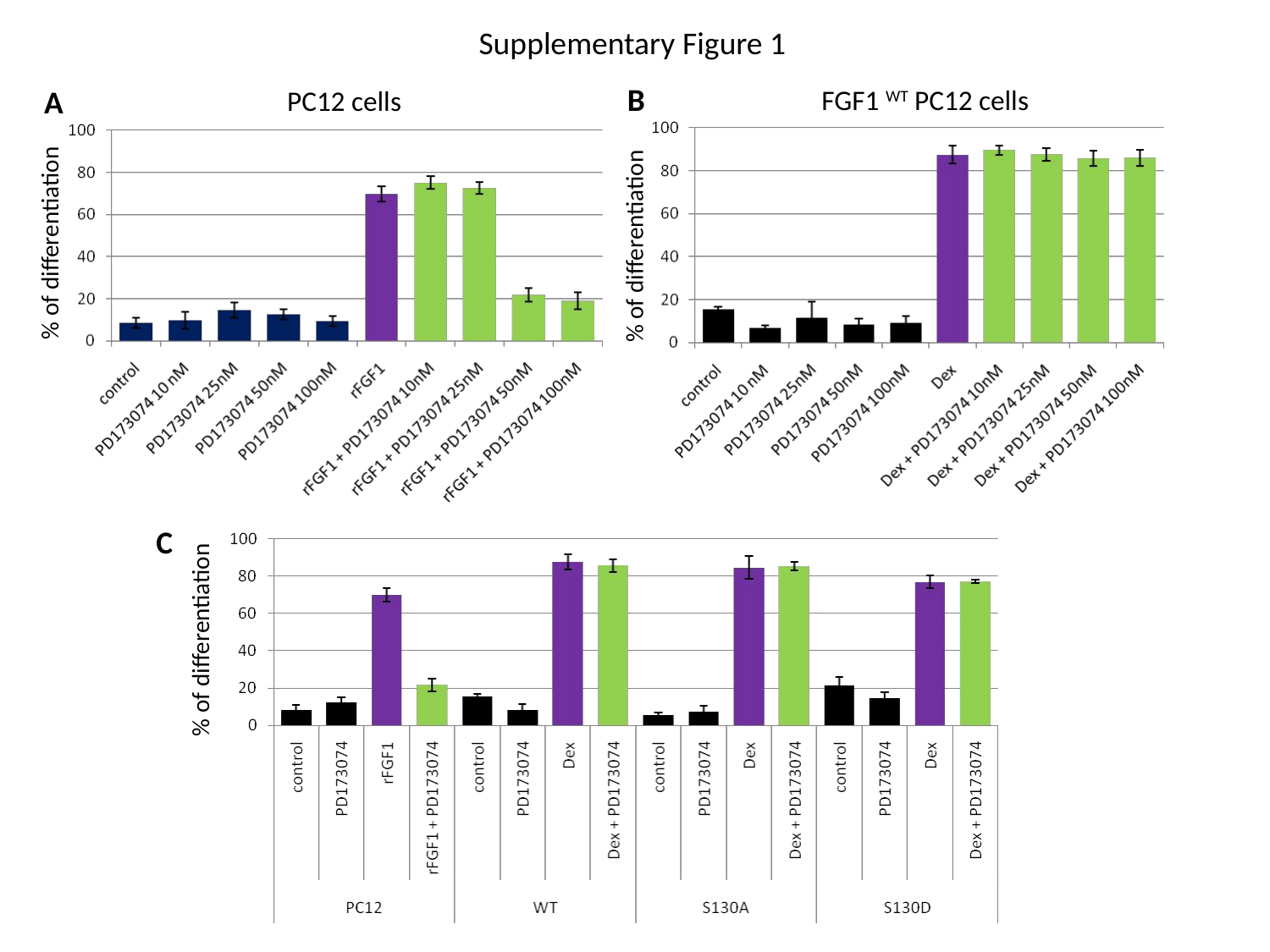

Supplementary Figure 1
B
A
FGF1 WT PC12 cells
PC12 cells
% of differentiation
% of differentiation
C
% of differentiation

## Slide 2
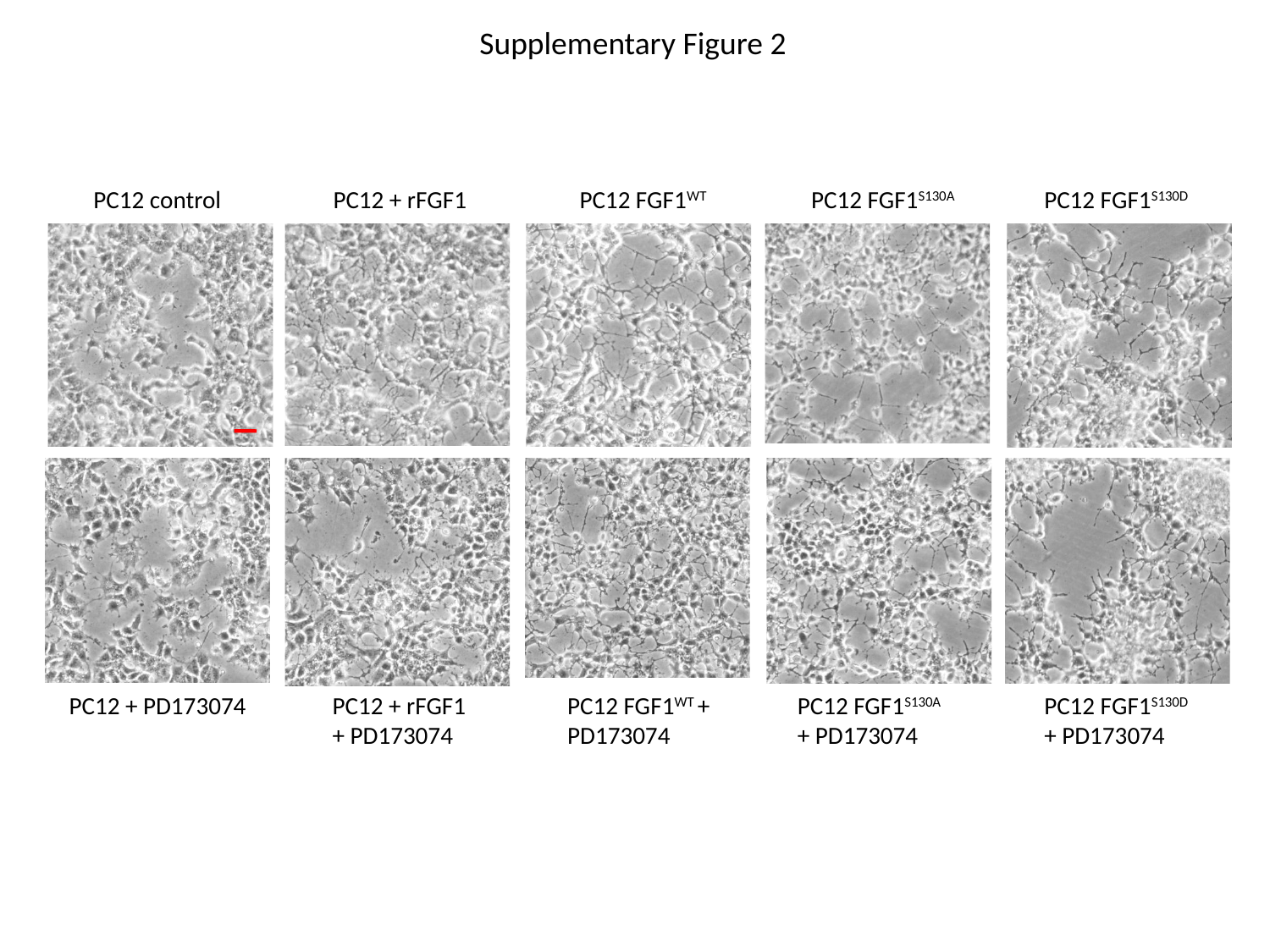

Supplementary Figure 2
PC12 control
PC12 + rFGF1
PC12 FGF1WT
PC12 FGF1S130A
PC12 FGF1S130D
PC12 + PD173074
PC12 + rFGF1 + PD173074
PC12 FGF1WT + PD173074
PC12 FGF1S130A + PD173074
PC12 FGF1S130D + PD173074

## Slide 3
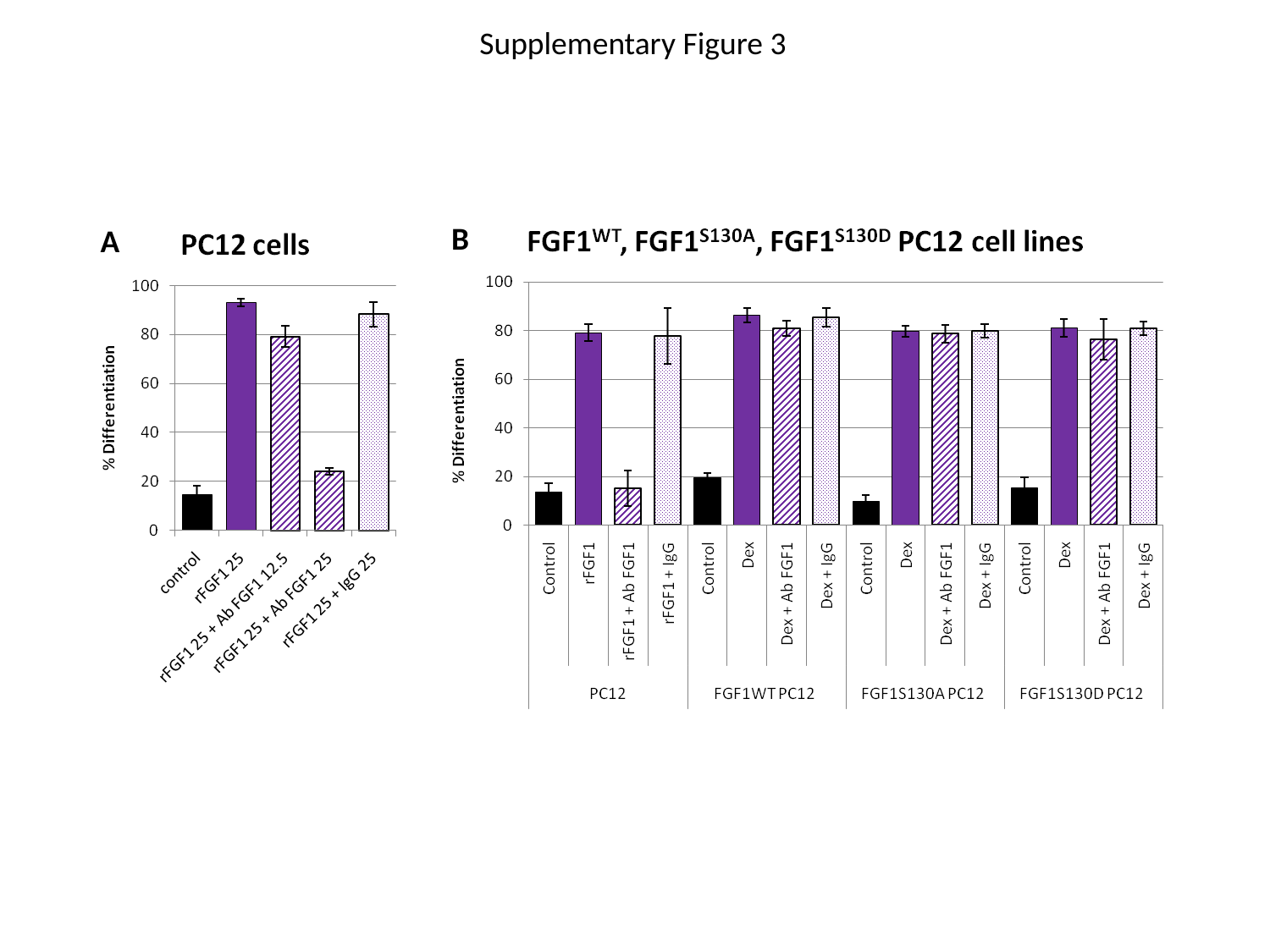

Supplementary Figure 3
B
A

## Slide 4
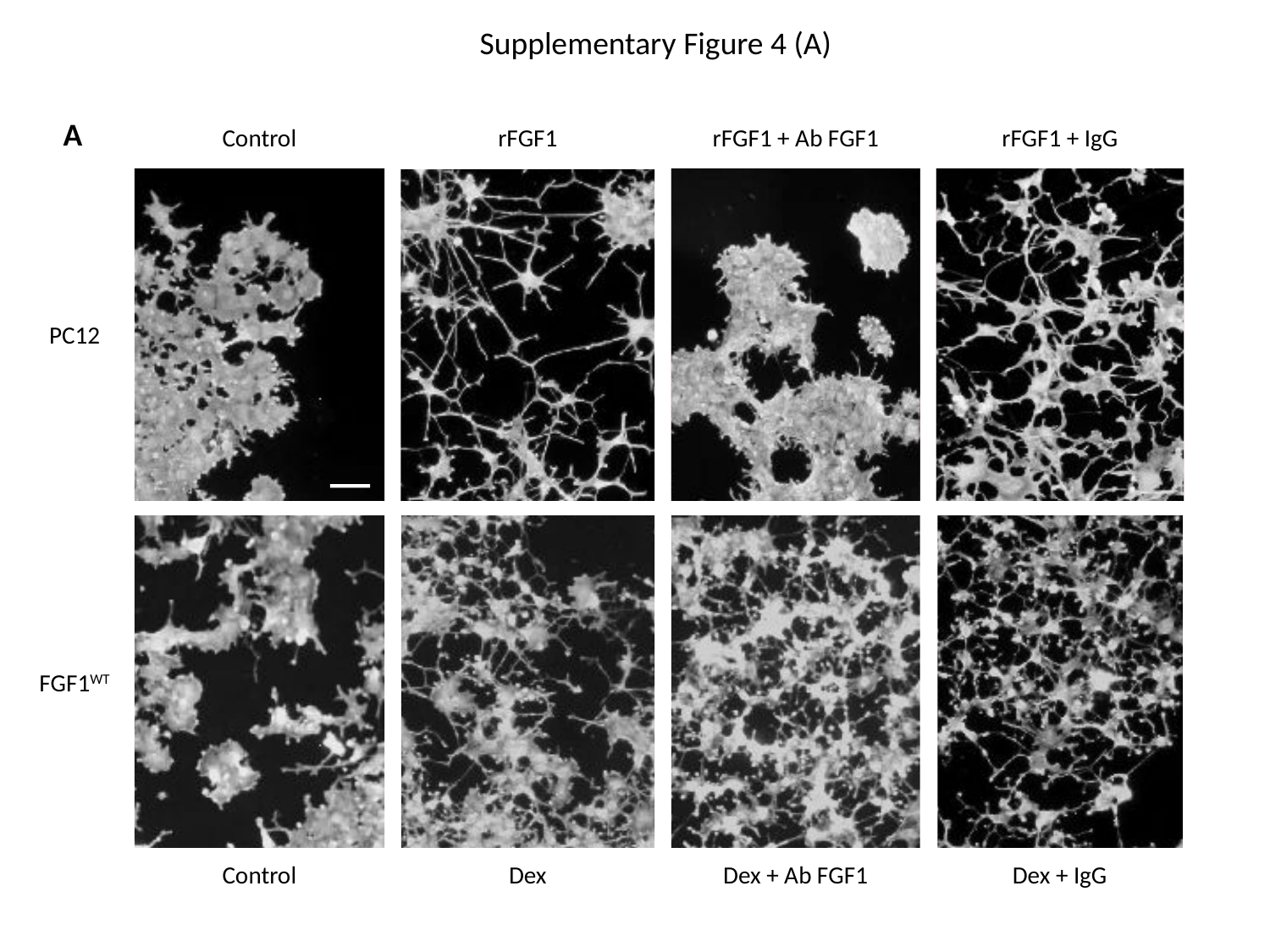

Supplementary Figure 4 (A)
A
Control
rFGF1
rFGF1 + Ab FGF1
rFGF1 + IgG
PC12
FGF1WT
Control
Dex
Dex + Ab FGF1
Dex + IgG

## Slide 5
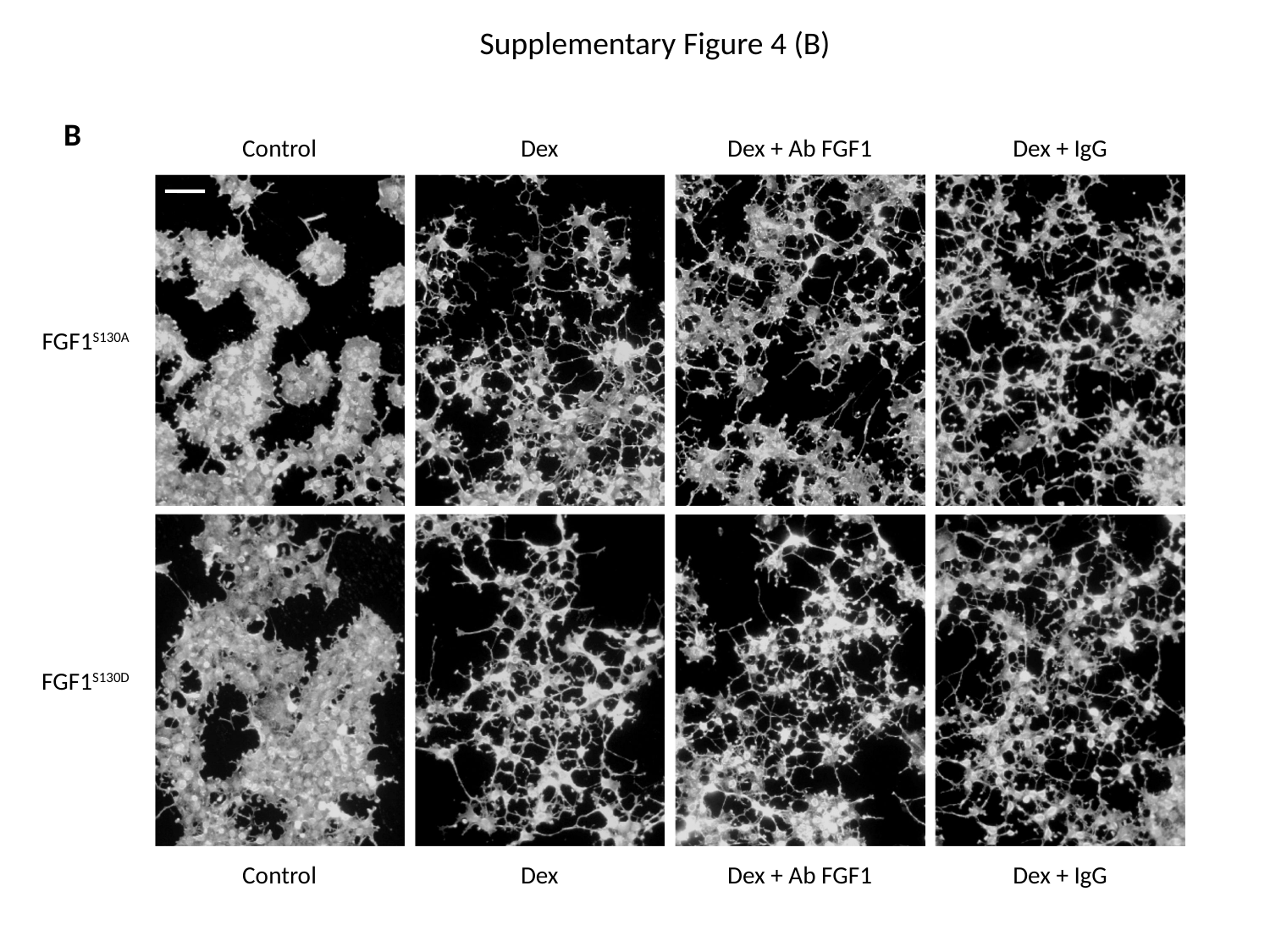

Supplementary Figure 4 (B)
B
Control
Dex
Dex + Ab FGF1
Dex + IgG
FGF1S130A
FGF1S130D
Control
Dex
Dex + Ab FGF1
Dex + IgG

## Slide 6
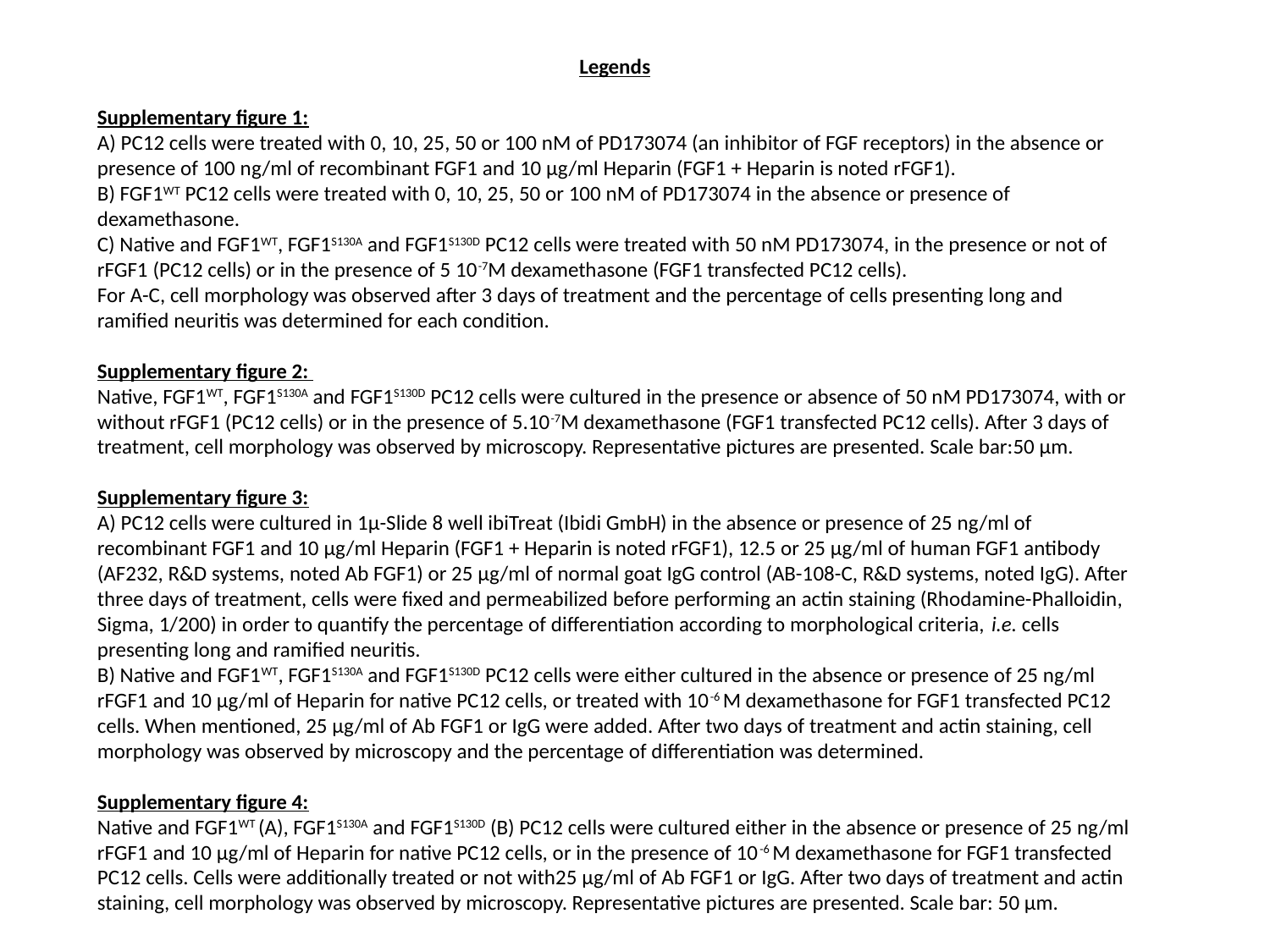

Legends
Supplementary figure 1:
A) PC12 cells were treated with 0, 10, 25, 50 or 100 nM of PD173074 (an inhibitor of FGF receptors) in the absence or presence of 100 ng/ml of recombinant FGF1 and 10 µg/ml Heparin (FGF1 + Heparin is noted rFGF1).
B) FGF1WT PC12 cells were treated with 0, 10, 25, 50 or 100 nM of PD173074 in the absence or presence of dexamethasone.
C) Native and FGF1WT, FGF1S130A and FGF1S130D PC12 cells were treated with 50 nM PD173074, in the presence or not of rFGF1 (PC12 cells) or in the presence of 5 10-7M dexamethasone (FGF1 transfected PC12 cells).
For A-C, cell morphology was observed after 3 days of treatment and the percentage of cells presenting long and ramified neuritis was determined for each condition.
Supplementary figure 2:
Native, FGF1WT, FGF1S130A and FGF1S130D PC12 cells were cultured in the presence or absence of 50 nM PD173074, with or without rFGF1 (PC12 cells) or in the presence of 5.10-7M dexamethasone (FGF1 transfected PC12 cells). After 3 days of treatment, cell morphology was observed by microscopy. Representative pictures are presented. Scale bar:50 µm.
Supplementary figure 3:
A) PC12 cells were cultured in 1µ-Slide 8 well ibiTreat (Ibidi GmbH) in the absence or presence of 25 ng/ml of recombinant FGF1 and 10 µg/ml Heparin (FGF1 + Heparin is noted rFGF1), 12.5 or 25 µg/ml of human FGF1 antibody (AF232, R&D systems, noted Ab FGF1) or 25 µg/ml of normal goat IgG control (AB-108-C, R&D systems, noted IgG). After three days of treatment, cells were fixed and permeabilized before performing an actin staining (Rhodamine-Phalloidin, Sigma, 1/200) in order to quantify the percentage of differentiation according to morphological criteria, i.e. cells presenting long and ramified neuritis.
B) Native and FGF1WT, FGF1S130A and FGF1S130D PC12 cells were either cultured in the absence or presence of 25 ng/ml rFGF1 and 10 µg/ml of Heparin for native PC12 cells, or treated with 10-6 M dexamethasone for FGF1 transfected PC12 cells. When mentioned, 25 µg/ml of Ab FGF1 or IgG were added. After two days of treatment and actin staining, cell morphology was observed by microscopy and the percentage of differentiation was determined.
Supplementary figure 4:
Native and FGF1WT (A), FGF1S130A and FGF1S130D (B) PC12 cells were cultured either in the absence or presence of 25 ng/ml rFGF1 and 10 µg/ml of Heparin for native PC12 cells, or in the presence of 10-6 M dexamethasone for FGF1 transfected PC12 cells. Cells were additionally treated or not with25 µg/ml of Ab FGF1 or IgG. After two days of treatment and actin staining, cell morphology was observed by microscopy. Representative pictures are presented. Scale bar: 50 µm.
